# Attenuation of Activated eIF2α Signaling by ISRIB Treatment After Spinal Cord Injury Improves Locomotor Function

**DOI:** 10.1007/s12031-021-01920-9

**Published:** 2021-10-13

**Authors:** Lei Chang, Xiangyang Liu, Jing Chen, Hongzhe Liu, Guoping Wang, Guohua Wang, Xiaoyun Liao, Xiongjie Shen

**Affiliations:** 1 Department of Spine Surgery, Hunan Provincial People’s Hospital, The First Affiliated Hospital of Hunan Normal University), No.61, West Jiefang Road, Changsha, 410005 China; 2 Department of Endocrinology, Hunan Provincial People’s Hospital, The First Affiliated Hospital of Hunan Normal University), Changsha, China; 3 Department of Anesthesiology, Hunan Provincial People’s Hospital, The First Affiliated Hospital of Hunan Normal University), Changsha, China

**Keywords:** Integrated stress response, eIF2α, Apoptosis, Locomotor function

## Abstract

Following spinal cord injury (SCI), multiple signaling cascades are activated instantaneously in the injured segments of the spinal cord to create a complex and pathogenic microenvironment, making it difficult to treat SCI. Nevertheless, the significance of the integrated stress response (ISR) to the series of physiological and pathological changes that occur after SCI remains unclear. Through western blotting (WB), we determined that the autophosphorylation of stress receptors (GCN2, PERK, PKR, and HRI) was enhanced after SCI, leading to increased phosphorylation of eIF2α at Ser51. Strikingly, we found that eIF2α was highly phosphorylated at 1 day post injury (dpi) and that this hypophosphorylation was maintained thereafter in the spinal cord, especially in neurons, which suggests that intervening with eIF2α phosphorylation may be a treatment strategy for SCI. Therefore, we employed the small molecule ISRIB, which inhibits eIF2α phosphorylation when the ISR is activated at moderate or low levels but not when the ISR is highly activated. Daily intraperitoneal injection of ISRIB significantly inhibited ISR signaling after SCI, reduced the cytosolic localization of RNA-binding proteins, and decreased neuronal apoptosis. Histological and functional experiments further demonstrated that treatment with ISRIB after SCI effectively curbed morphological deterioration and promoted the recovery of locomotor function. In summary, the ISR plays an important role in SCI, and ISRIB is a promising drug for the treatment of SCI.

## Introduction

As one of the most serious public health problems worldwide, spinal cord injury (SCI) has serious physical and psychological impacts and imposes a heavy burden on society (Rubiano et al. 2015; Wagner et al. 2018). Since neurons in the mammalian central nervous system (CNS) cannot regenerate, the treatment of CNS injury is a challenge (Griffin 2003; Miller et al. 2016). In addition to irreversible necrosis caused by transient injury, secondary pathophysiological changes resulting from injury, such as inflammation (Tsarouchas et al. 2018), oxidative damage (Wang et al. 2019), and proteostasis imbalance (Valenzuela et al. 2012), can worsen the injury and increase mortality by causing loss of neurons and dysfunction of neural network connections. Therefore, a large number of studies have focused on secondary injury after SCI to identify potential therapeutic targets to curb the progression of the disease.

The integrated stress response (ISR) is a conservative defense response (Kroemer et al. 2010; Costa-Mattioli and Walter 2020; Kong et al. 2020) to proteostasis defects, nutritional deprivation, viral infection, redox imbalance, etc. The initiation of the ISR depends on the phosphorylation of Ser51 in eIF2α, which is induced by PERK, PKR, GCN2, and HRI (Malvezzi et al. 2020). Normally, the eIF2 ternary complex (TC), which consists of eIF2 (including the α, β, and γ subunits), GTP, and Met-tRNAi, participates in elongation during the translation of genes starting with AUG (Costa-Mattioli and Walter 2020). Phosphorylated eIF2α can serve as a noncompetitive inhibitor of eIF2B to inhibit eIF2B-catalyzed dissociation of GDP from inactivated eIF2, thus blocking global protein translation. The transient inhibition of protein translation is conducive to mobilizing the cell’s armory against stress, while chronic inhibition contributes to decreasing neurogenesis and impairing neuronal activity (Surget et al. 2011). In addition, the phosphorylation of eIF2α can trigger the nuclear translocation of ATF4 and then promote the expression of stress resistance genes or proapoptotic genes (Zhou et al. 2020).

Appropriate phosphorylation of eIF2α contributes to preserving cellular homeostasis in the context of cellular stress by limiting protein synthesis and promoting the translation of specific mRNAs to produce proteins involved in autophagy, the unfolded protein response (UPR), and heat shock (Zhu et al. 2019). Additionally, the expression of the apoptotic gene CHOP can be upregulated to induce apoptosis of damaged cells when activation of the ISR fails to resolve stress (Liu and Ye 2011; Arai et al. 2020). Nevertheless, existing evidence has demonstrated that the proteostasis imbalance-induced endoplasmic reticulum stress response (ERSR) can significantly affect the fate of neurons after SCI (Wang et al. 2017; Li et al. 2019), but the role and underlying mechanism of the ISR after SCI remain unclear.

Considering the dual role of the ISR in stress relief and apoptosis promotion, the significance of the ISR after SCI should be urgently explored. Here, we employed the small molecular compound ISRIB, which can cross the blood-brain barrier and promote the assembly of eIF2B to catalyze the dissociation of GDP from the inactivated TC to inhibit the ISR signaling pathway (Zyryanova et al. 2018; Rabouw et al. 2019). Since ISRIB works by promoting eIF2B assembly, it fails to inhibit ISR when unassembled eIF2B is consumed by highly phosphorylated eIF2α. Thus, the ISR is not inhibited by ISRIB when it is overactivated, while the ISR is blocked by ISRIB when it is activated at a moderate or low level. Therefore, whether the application of ISRIB is beneficial for the survival of neurons after secondary injury and the improvement of behavioral function after SCI remains to be studied.

## Materials and Methods

### Animal and Ethic Statement

A total of 78 healthy male C57BL/6JNifdc mice (8-week-old, 20––23 g) were used in our study. The mice are housed in a clean grade environment with 12 h light and dark cycle (23 °C, 60% humidity), and they have free access to water and food. All experimental procedures were approved by Medical Ethics Committee of Hunan People’s Hospital (First Affiliated Hospital of Hunan Normal University, 2019-41) and were performed in accordance with the National Institutes of Health guide for the care and use of Laboratory animals.

### Regents and Antibodies

Anti-PKR (Cat. No. 18244-1-AP), anti-ATF4 (Cat. No. 10835-1-AP), anti-histone H3 (Cat. No. 17168-1-AP), anti-TIA-1 (Cat. No. 12133-2-AP), anti-FUS (Cat. No. 11570-1-AP), anti-phospho-eIF2S1 (Ser51) (Cat. No. 28740-1-AP), anti-eIF2α (Cat. No. 11170-1-AP), anti-GAPDH (Cat. No. 60004-1-Ig), anti-GADD34 (Cat. No. 10449-1-AP), and anti-CHOP (Cat. No. 15204-1-AP) antibodies were purchased from Proteintech (Chicago, USA). Anti-GCN2 (Cat. No. PA5-105886) antibody was purchased from Cell Signaling Technology (Danvers, MA, USA). Anti-caspase 12 antibody (Cas12, Cat. No. 3282-100) was obtained from BioVision (San Francisco, USA). Anti-PERK (Cat. No. SC377400), anti-HRI (Cat. No. SC-365239), and anti-TDP-43 (Cat. No. SC-376311) antibodies were purchased from Santa Cruz Biotechnology (Dallas, TX, USA). Anti-phospho-PERK (Thr980) (Cat. No. BS-3330R) antibody was purchased from Bioss (Beijing, China). Anti-puromycin (Cat. No. MABE343) antibody and puromycin (Cat. No. 540222) were obtained from Merck (Darmstadt, Germany). Anti-phospho-GCN2 (Thr899) (Cat. No. PA5-105886) and anti-phosphor-PKR (Thr446 + Thr451) (Cat. No. BS-3337R) antibodies were obtained from Thermo Fisher Scientific (Waltham, MA, USA). Anti-NeuN (Cat. No. ab104224) and anti-cleaved caspase-3 (C-Cas3, Cat. No. ab49822) antibodies, Alexa Fluor 488-labeled or Alexa Fluor 647-labeled goat anti-Rabbit or mouse secondary antibodies, and Bradford (Cat. No. ab119216) were purchased from Abcam (Cambridgeshire, England). Goat anti-rabbit/mouse IgG (H + L) HRP were obtained from MultiSciences (Hangzhou, Zhejiang, China). DAPI-containing fluorescent mounting media (Cat. No. 36308ES20) were purchased from YEASEN (Shanghai, China). Hematoxylin and eosin (H&E) staining kit (Cat. No. G1120) was obtained from Solarbio (Beijing, China). Loading buffer (Cat. No. P0015L), antigen repair solution (Cat. No. P0083), and protein inhibitors (Cat. No. P1005) were obtained from Beyotime (Shanghai, China). Lysis buffer (Cat. No. AR0101) was purchased from BOSTER (Wuhan, Hubei, China). Bovine serum albumin (Catalog No. A7030) was purchased from Sigma (Shanghai, China).

### SCI Model and Drug Treatment

Mice receiving SCI and sham surgery were anesthetized with 4% isoflurane and then maintained with 1.5% isoflurane. For the SCI group, after the laminectomy, the exposed T9 segment of spinal cord suffered moderate contusion (10 g, 1.5 cm) as previously described (Liu et al. 2015, 2018), while the sham group only received laminectomy. Heating pads were applied to maintain body temperature (37 °C) during and after surgery. After SCI, bladders were manually expressed twice daily until a reflex bladder was established (12–16 days after SCI). ISRIB (Cat. No. HY-12495, MedChemExpress, Monmouth Junction, USA) was prepared and injected intraperitoneally (2.5 mg/kg/day) into mice after SCI as previously described (Krukowski et al. 2020).

### BMS Scoring System and Footprint Analysis

In order to assess the recovery of locomotion function of injured mice, BMS scoring was conducted at 0, 1, 3, 7, 14, and 28 days post injury (dpi) as previously described (Wang et al. 2018). Briefly, the mice were placed in an open field with a diameter of 42 inches and observed for 4 min by a well-trained observer who was blind to the experimental group. The scoring range is 0–9 (0, complete hind limb paralysis; 9, normal exercise). This is based on hind limb movement in an open field, including hind limb joint movement, weight support, plantar stepping, coordination, paw position, trunk, and tail control. After the BMS scoring, the mice hind limbs were immersed in red ink, and then the movement track of the mice was collected on the white runway for footprint analysis. Six independent mice were used for both foot imprinting and BMS scoring, and each mouse was tested 3 times to ensure accuracy.

### Tissue Preparation and Histopathologic Analysis

At the indicated time point (1, 3, 7, and 28 dpi), the mice were sacrificed by carbon dioxide inhalation. For western blotting (WB) analysis, after the sacrificed mice were perfused with saline in the left ventricle, a total of 1 cm of spinal cord tissue above and below the epicenter was obtained. For immunofluorescence (IF) staining and H&E staining, after the sacrificed mice were perfused with paraformaldehyde (4%) in the left ventricle, a total of 1 cm of spinal cord tissue above and below the epicenter was immersed in paraformaldehyde (4%) for 48 h and then dehydrated and embedded in paraffin. Transverse sections of spinal cord (5 μm) were obtained and loaded on glass slides. H&E staining was performed according to the guidance of its manual after footprint analysis (Cat. No. G1120, Solarbio). The tissue section samples of each group in H&E were derived from 6 independent mice.

### Extraction of Nuclear Proteins and Cytoplasmic Proteins

To determine the distribution of transcription factor ATF4 and stress granules (SGs) associated proteins (TIA-1, FUS, and TDP-43) in the nucleus and cytoplasm, the nuclear proteins and cytoplasmic proteins in spinal cord tissue obtained above at 7 dpi were extracted by a nuclear protein and cytoplasmic protein extraction kit (Cat. No. P0027, Beyotime). The purity of the extracted nuclear components and cytoplasmic components were determined by detecting the expression levels of histone H3 (nuclear protein) and GAPDH (cytoplasmic protein).

### Measurement of Protein Synthesis

In order to define the variations of protein synthesis around epicenter of spinal cord after SCI, puromycin was applied to label nascent peptides. Before being sacrificed, the mice were injected intraperitoneally with 0.04 mM/kg puromycin for 30 min as previously described (Park et al. 2014). Next, the expression level of puromycin-labeled proteins around epicenter of spinal cord was detected through WB analysis with anti-puromycin antibody.

### WB Analysis

The spinal cord tissue obtained above for total proteins and puromycin-labeled proteins measurement were homogenized and lysed at 4 °C in the presence of lysis buffer and protease inhibitors. Bradford was subsequently used to measure the concentration of protein in tissue lysates. A total of 40 μg of protein was separated by electrophoresis on sodium dodecyl sulfate-polyacrylamide gel in the presence of loading buffer and then transferred to polyvinylidene fluoride membrane (Cat. No. 1620256, Bio-Rad, Berkeley, CA, USA). Next, the membranes were blocked with skimmed milk (5%) for 1 h at room temperature, followed by incubated with specific primary antibodies overnight at 4 °C. Subsequently, the membranes were incubated with horseradish peroxidase-conjugated secondary antibodies at room temperature for 1 h. ChemiDicTM XRS+ Imaging System (Bio-Rad, Berkeley, CA, USA) was applied to visualized signals, and the signals were quantified by the ImageJ software. The protein samples of each group in WB came from 3 independent mice, and the samples of each mouse were tested three times to obtain the average value.

### IF Staining

The sections (1, 3, and 7 dpi) obtained above were dewaxed and gradient hydrated and then subjected to high-pressure antigen repair in the presence of antigen retrieval solution. The sections were then blocked with bovine serum albumin (5%) at room temperature for 1 h, followed by incubating overnight at 4 °C with specific antibodies. Next, the sections were incubated with Alexa Fluor 488-labeled or Alexa Fluor 647-labeled goat anti-rabbit or mouse antibodies for 1 h at 37 °C and mounted with DAPI-containing fluorescent mounting media. In order to eliminate the misleading caused by non-specific staining, we added a negative control group (only incubate the secondary antibody of the corresponding species without incubating the primary antibody) in each experiment to ensure the specificity of the antibodies. Zeiss LSM710 microscope was used to capture images. In order to analyze the changes in fluorescence intensity, we first determined the range of neuron cell bodies through the NeuN channel and then employed ImageJ to determine the area of this range and measure the total fluorescence intensity of p-eIF2α or C-Cas3 in this area. The average fluorescence intensity is determined by calculating the ratio of the sum of the fluorescence intensity to the area, which is then used for statistics.

### Statistical Analysis

All data were expressed as the mean ± SEM. The normality of collected data was first determined by Shapiro-Wilk test. Then, the significant difference among multiple groups was measured by one-way analysis of variance (ANOVA) test, followed by Tukey’s multiple comparison test to exhibit the difference between each two groups. The significant difference between SCI group and SCI+ISRIB group in BMS scoring was measured by Mann-Whitney test. The statistical evaluation of our data was performed in GraphPad Prism 8 Software. *P* < 0.05 was considered statistically different. Except for BMS scoring and footprint, other experiments were repeated three times independently to ensure accuracy.

## Results

### Activation of the ISR in Spinal Cord After SCI

**Fig. 1:**
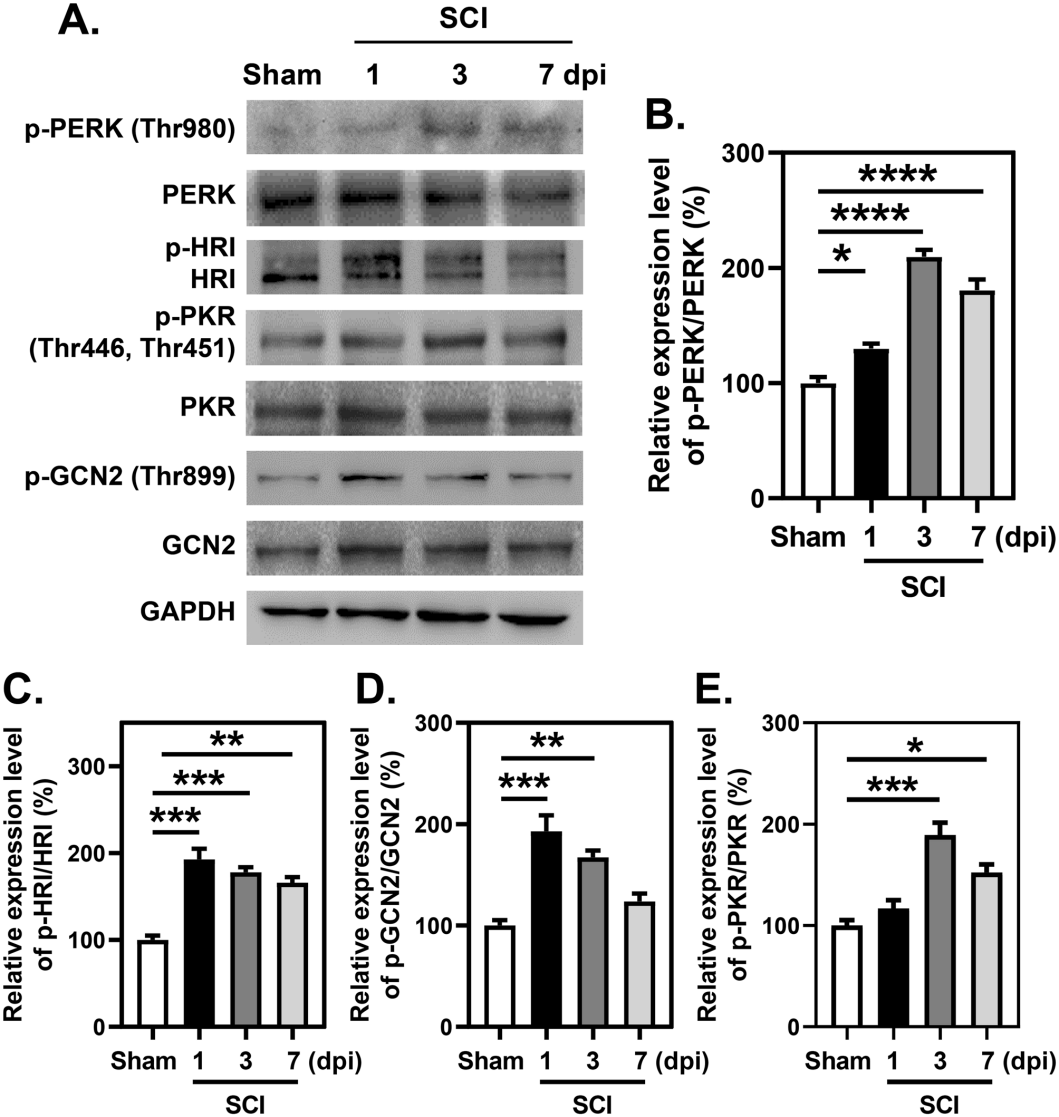
Activation of ISR in spinal cord after SCI. **A**–**E** WB analysis and quantitative analysis of phosphorylation of PERK, HRI, GCN2, and PKR at indicated time points following SCI. *n*=3 (each group contains 3 mice). **B**–**E** One-way ANOVA and Tukey’s multiple comparisons test. *P < 0.05, sham vs. 1 dpi, sham vs. 7 dpi; **P < 0.01, sham vs. 3 dpi, sham vs. 7 dpi; ***P <0.001, sham vs. 1 dpi, sham vs. 3dpi; ****P < 0.0001, sham vs. 3 dpi, sham vs. 7 dpi

To define the significance of the ISR after SCI, the activation of four arms of the ISR, i.e., PERK, GCN2, HRI, and PKR, was assessed by determining the phosphorylation state of these proteins at 1, 3, and 7 days post injury (dpi). Western blotting (WB) revealed that phosphorylation of PERK at Thr980 and the phosphorylation of PKR at Thr446 and Thr451 were significantly increased after SCI and peaked at 3 dpi (Fig. 1A, B, and E). Additionally, the phosphorylation of HRI and the phosphorylation GCN2 at Thr899 were also enhanced after SCI, but they peaked at 1 dpi (Fig. 1A, C, and D). These data suggest that the ISR is activated in the injured segments of the spinal cord.

### Persistent Activation of the ISR in Neurons After SCI

**Fig. 2:**
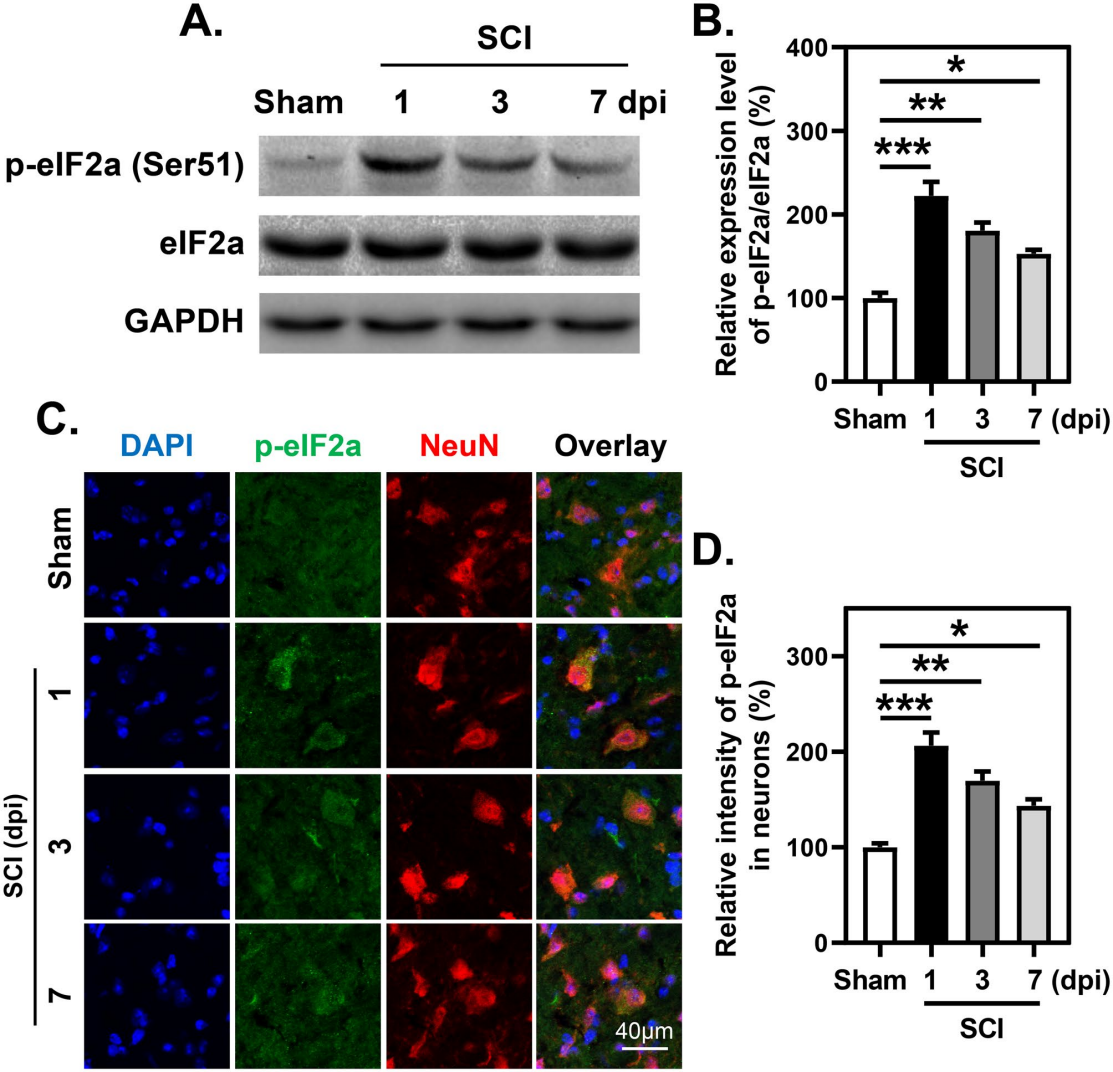
Phosphorylation of eIF2α in spinal cord and neurons after SCI. **A** and **B** WB analysis and quantitative analysis of phosphorylation of eIF2α. *n*=3. **C** and **D** Representative images and quantitative analysis of spinal cord transection stained with anti-p-eIF2α and anti-NeuN antibodies at indicated time points following SCI. *n*=3. **B** and **D** One-way ANOVA and Tukey’s multiple comparisons test. *P < 0.05, sham vs. 7 dpi; **P < 0.01, sham vs. 3 dpi; ***P <0.001, sham vs. 1 dpi

Next, the initiation of downstream signaling of these four arms was detected by WB and IF staining. The phosphorylation of eIF2α at Ser51 is the core event of ISR activation. Here, the results revealed a significant increase in the phosphorylation level of eIF2α observed at 1 dpi, and there was still a significant difference at 7 dpi (Fig. 2A, B). Especially in NeuN-positive cells (neuron), the phosphorylation level of eIF2α was still significantly higher in the SCI group than in the sham group at 7 dpi (Fig. 2C, D), revealing that the ISR was activated around the epicenter of the spinal cord and that ISR activation was not temporary after SCI.

### ISRIB Treatment Reverses SCI-Induced Apoptosis

**Fig. 3:**
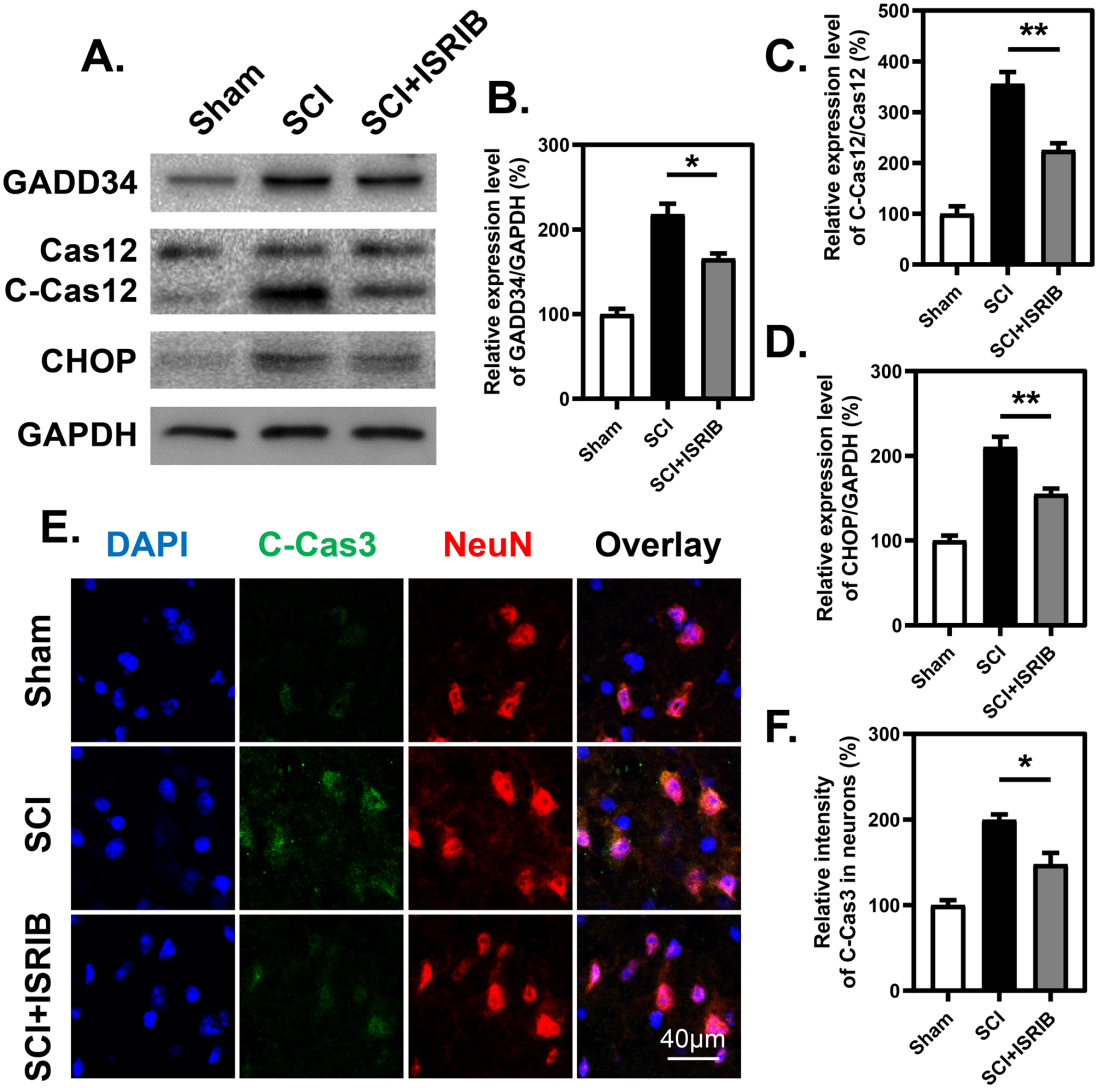
ISRIB inhibits ISR signal and apoptosis after SCI. **A**–**D** WB analysis and quantitative analysis of ISR-related proteins (GADD34 and CHOP) and proteotoxicity-associated apoptotic protein cleaved-caspase 12 (C-Cas12) at 7 dpi with or without treatment of ISRIB. *n*=3. **E** and **F** Representative images and quantitative analysis of spinal cord transection stained with anti-cleaved-caspase 3 (C-Cas3) and anti-NeuN antibodies at 7 dpi with or without treatment of ISRIB. *n*=3. **B**–**D** and **F** One-way ANOVA and Tukey’s multiple comparisons test. *P < 0.05, **P < 0.01, SCI vs. SCI + ISRIB

The ISR inhibitor ISRIB was then employed to better determine the significance of the ISR after SCI. WB revealed that daily administration of ISRIB decreased the expression level of the eIF2α downstream protein GADD34 and the apoptotic protein CHOP and attenuated the cleavage of the proteotoxicity-associated apoptotic protein caspase 12 (Cas12) in the spinal cord at 7 dpi (Fig. 3A–D). In addition, IF staining results proved that the cleavage of the classic apoptotic protein caspase 3 in neurons was reduced in the ISRIB-treated group compared with the SCI group at 7 dpi (Fig. 3E, F), indicating that ISRIB can limit the apoptotic cascade after SCI.

### SCI-Induced Protein Translocation and Inhibition of Protein Synthesis Are Reversed by ISRIB Treatment

**Fig. 4:**
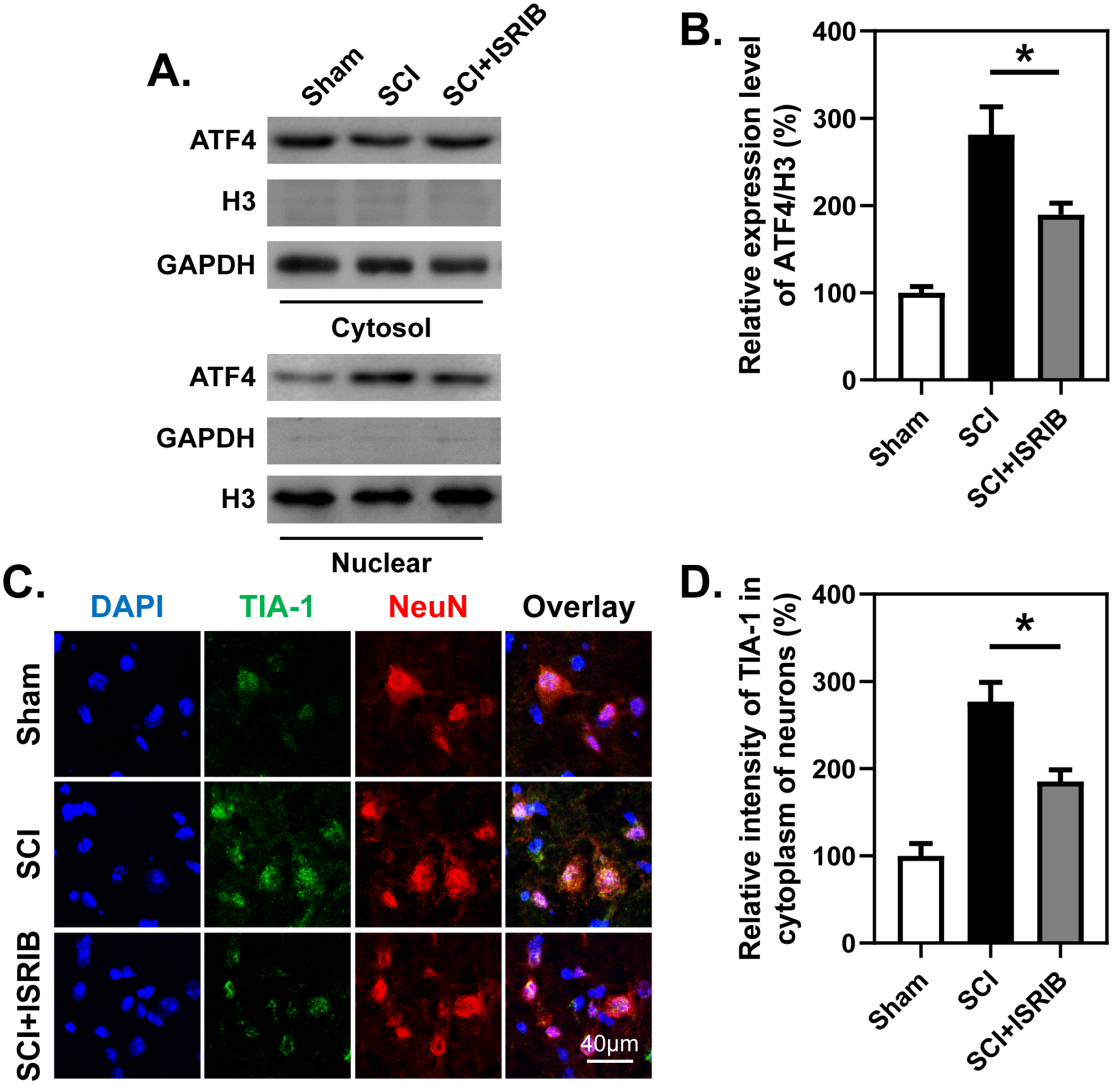
ISRIB decreases nuclear translocation of ATF4 and formation of stress granules after SCI. **A** and **B** WB analysis and quantitative analysis of nuclear translocation of ATF4 at 7 dpi with or without treatment of ISRIB. *n*=3. **C** and **D** Representative images and quantitative analysis of spinal cord transection stained with anti-TIA-1 and anti-NeuN antibodies at 7 dpi with or without treatment of ISRIB. *n*=3. **B** and **D** One-way ANOVA and Tukey’s multiple comparisons test. *P < 0.05, SCI vs. SCI + ISRIB

**Fig. 5:**
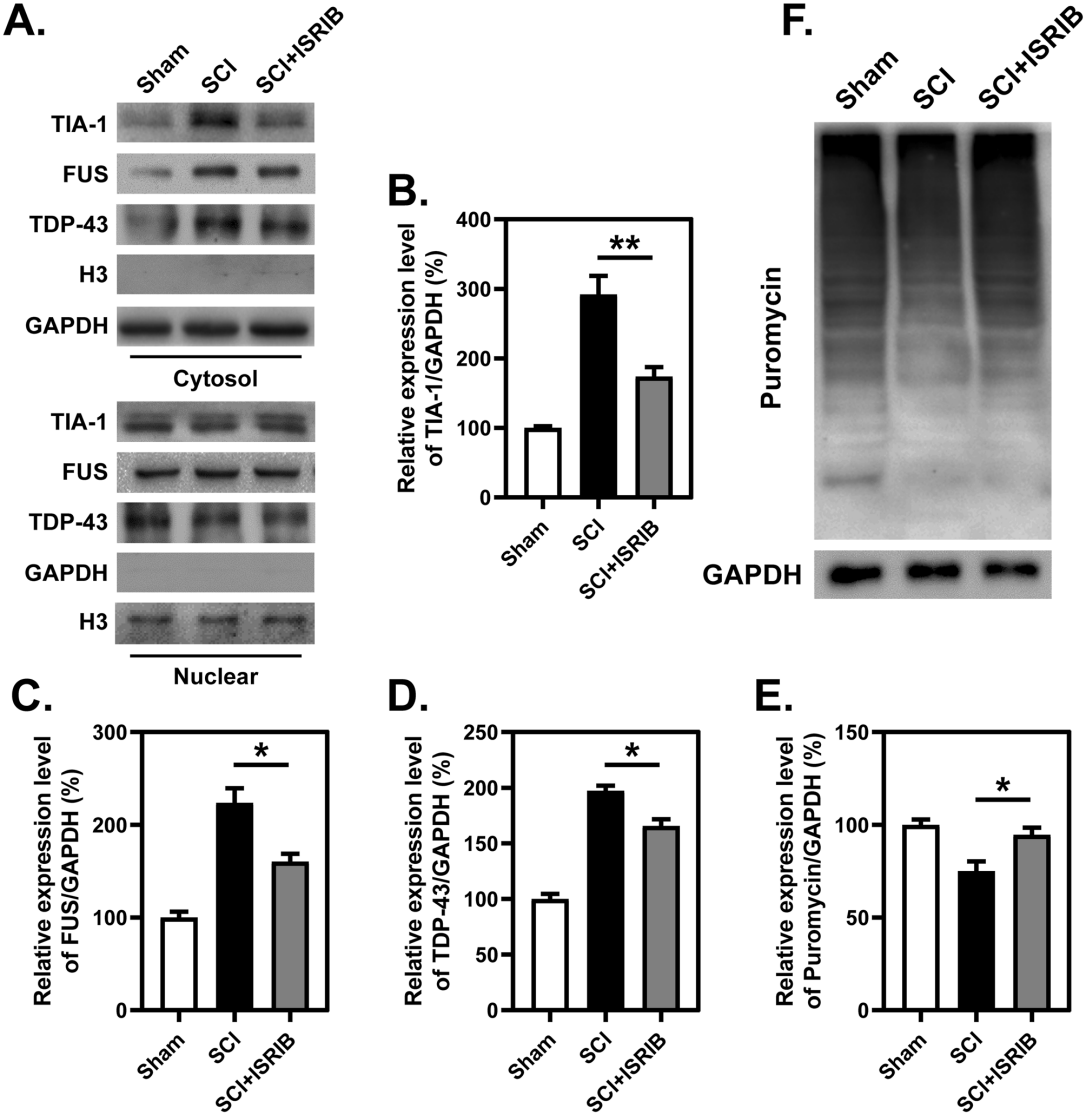
ISRIB dwindles cytosolic location of RNA-binding proteins and enhances protein synthesis after SCI. **A**–**D** WB analysis and quantitative analysis of nuclear translocation of TIA-1, FUS, and TDP-43 at 7 dpi with or without treatment of ISRIB. *n*=3. **E** and **F** WB analysis and quantitative analysis of puromycin-labeled newly synthesis peptides at 7 dpi with or without treatment of ISRIB. *n*=3. **B**–**E** One-way ANOVA and Tukey’s multiple comparisons test. *P < 0.05, **P < 0.01, SCI vs. SCI + ISRIB

Subsequently, nuclear and cytoplasmic proteins were isolated from spinal cord tissues, and it was found that the translocation of ATF4 from the cytosol to the nucleus was enhanced after SCI but that ISRIB administration significantly inhibited this translocation (Fig. 4A, B). Detection of newly synthesized puromycin-labeled peptides also revealed that protein translation, another process involving eIF2α phosphorylation, was impaired after SCI, but this impairment was alleviated by ISRIB treatment after SCI (Fig. 5E, F). The assembly of stress granules (SGs) in the cytoplasm was evaluated by IF staining for TIA-1 and analysis of the cytosolic translocation of TIA-1, FUS, and TDP-43 after SCI by WB. It was found that ISRIB treatment significantly decreased the cytosolic localization of SG assembly associated proteins in the spinal cord, especially in neurons, after SCI (Figs. 4C, D and 5A–D).

### ISRIB Treatment Ameliorates Pathological Deterioration and Improves Locomotor Function After SCI

**Fig. 6:**
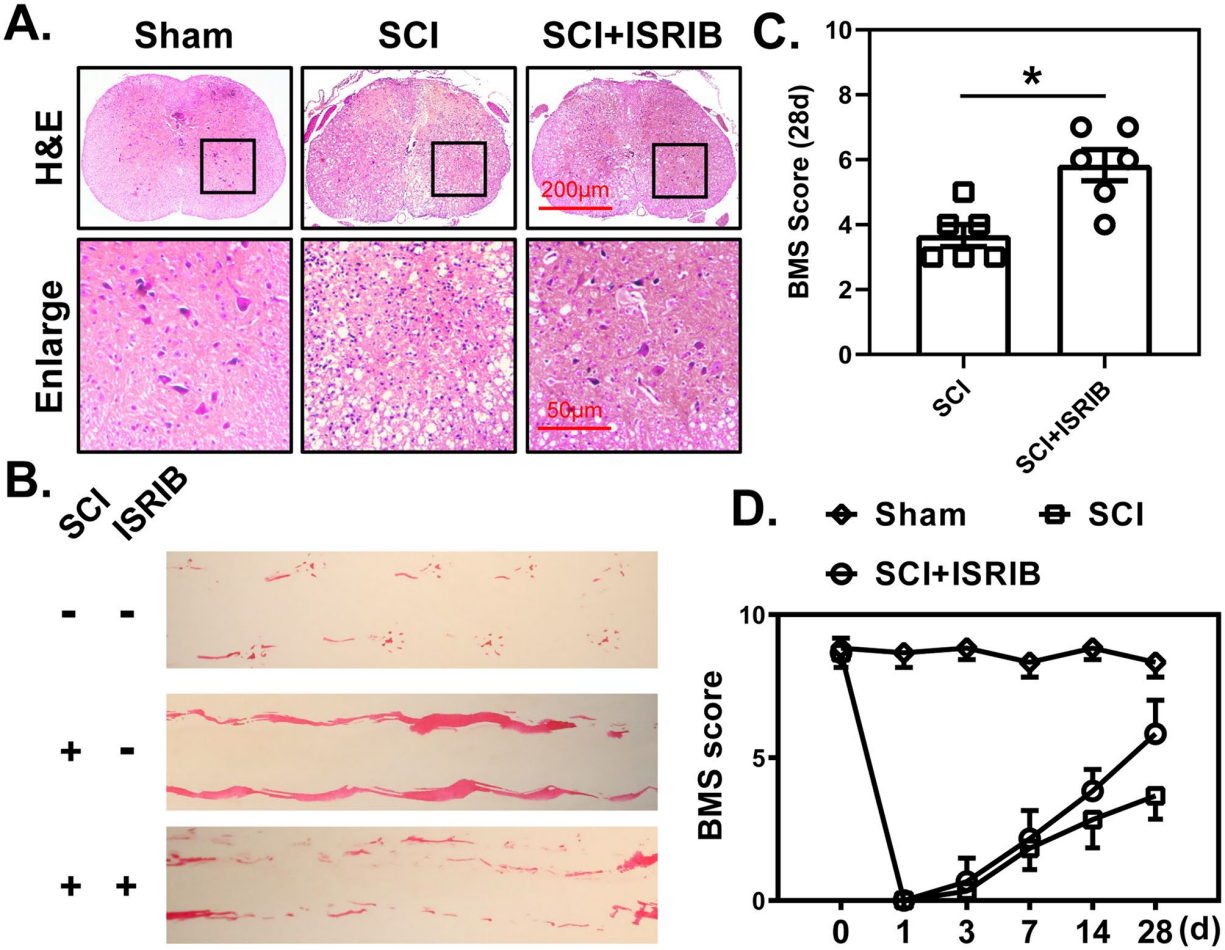
ISRIB improves histopathology and locomotor function after SCI. **A** Representative images of spinal cord transection stained with H&E at 28 dpi. *n*=6. **B** Representative images of locomotion trajectories of mice hind limbs. *n*=6. **C** and **D** Quantitative analysis of BMS score from 0–28 dpi and at 28 dpi among different groups. *n*=6. Mann-Whitney test. *P < 0.05, SCI vs. SCI + ISRIB

H&E staining, footprint analysis, and the BMS scoring system were then used to evaluate the therapeutic effects of ISRIB treatment after SCI. H&E staining revealed that ISRIB treatment reduced the loss of neurons in the anterior horn and the formation of tissue cavities around the epicenter of the spinal cord at 28 dpi (Fig. 6A). Footprint analysis showed that ISRIB treatment improved the locomotor function of the hind limbs at 28 dpi, as indicated by an improved trajectory (Fig. 6B). In addition, the recovery of behavioral function was promoted by ISRIB administration after SCI, and the BMS scores of ISRIB-treated mice were significantly higher than those of untreated mice at 28 dpi (Fig. 6C, D).

## Discussion

The continuous expansion of secondary injury after SCI, as an incurable disease, greatly affects later functional recovery. Current therapeutic strategies for SCI mainly involve the following: (1) attenuation of neuronal loss; (2) promotion of neuronal network reconstruction; and (3) stem cell transplantation (Guo et al. 2012; Das et al. 2013; Lai et al. 2013; Sabelstrom et al. 2013). Studies have highlighted that the number of neurons in the injured segment of the spinal cord determines functional recovery after SCI. Thus, we aimed to inhibit apoptotic cascades in neurons and improve neuronal function after SCI.

Previous studies have verified that perturbation of ER homeostasis, which triggers rapid ERSR and is involved in morphological deterioration, occurs in different SCI models (Valenzuela et al. 2012; Ohri et al. 2013; Liu et al. 2015). In addition, overactivation of oxidative stress caused by excessive generation of reactive oxygen species and ischemia/hypoxia caused by blood vessel loss have been demonstrated to be involved in secondary degeneration following SCI and may be therapeutic targets (Li et al. 2017; Wang et al. 2020a, b). Nevertheless, the role of the ISR, which responds to multiple stresses after SCI, remains to be clarified. Here, we found that the four branches of the ISR (PERK, HRI, GCN2, PKR) were all activated after SCI, as indicated by increased autophosphorylation of each protein. In addition, the activation peaks of the four branches exhibited incomplete overlap in our study, indicating the complexity of the microenvironment after SCI.

Subsequently, it was determined that the ISR downstream molecule eIF2α was activated after SCI, and the results showed that the phosphorylation of eIF2α at Ser51 was enhanced after SCI. Strikingly, eIF2α phosphorylation peaked at 1 dpi, and then eIF2α activation was maintained a low level, suggesting that the ISR was strongly stimulated in the acute phase and then continued to be activated in the subacute phase after SCI. It is widely accepted that transient activation of the ISR can lead to reprogramming of protein transcription and regulate gene expression in response to stress and resist stress, while persistence and abnormal activation of the ISR can affect neural function and even trigger cellular apoptosis by limiting the translation of proteins required for neural function (Fan et al. 2013; Lopez-Erauskin et al. 2018; Kapur et al. 2020). Therefore, strategies that can maintain ISR-induced stress resistance in the acute phase and prevent ISR-induced neurodegeneration in the subacute phase after SCI are promising.

Thus, the ISR inhibitor ISRIB is a therapeutic candidate due to its unique target. ISRIB serves as an activator of eIF2B, and the assembly of eIF2B can be promoted under moderate stress to reactivate eIF2 and thus restore global translation (Costa-Mattioli and Walter 2020). Recent studies have highlighted the significance of ISRIB in enhancing cognition and ameliorating cognitive deficits after brain injury (Tsai et al. 2018), but its role in other types of nerve injuries and neurodegenerative diseases needs to be further determined. After administration of ISRIB to mice subjected to SCI, eIF2α downstream signaling was significantly suppressed, as characterized by decreased translocation of ATF4 to the nucleus and subsequent downregulation of GADD34 and CHOP expression. By labeling nascent peptide chains with puromycin, we found that protein translation was decreased after SCI and that protein translation was promoted after ISRIB administration, confirming that the decrease in protein translation after SCI was attributed to redistribution of translation caused by eIF2α phosphorylation and nuclear translocation of its downstream molecule ATF4.

As a result of multiple stresses and obstacles to protein translation, SGs can be assembled as a result of the translocation of RNAs and RNA-binding proteins to the cytoplasm to arrest mRNA and then inhibit translation (Nahm et al. 2020). It has been reported that the formation of SGs is induced in the brain after traumatic brain injury (TBI), another type of CNS injury, and that SGs might aggregate. Genetic and pharmacological induction of SG clearance alleviates neural degeneration after TBI (Anderson et al. 2018). Our results demonstrated for the first time that SG assembly related RNA-binding proteins were also abundant in the cytoplasm in the spinal cord, especially in neurons, in the subacute phase after SCI, suggesting delayed clearance of SGs after SCI. Nevertheless, ISRIB treatment reduced the levels of RNA-binding proteins in the cytoplasm and even the expression levels of apoptosis-associated genes at 7 dpi, indicating that ISRIB contributes to inhibiting the SCI-triggered cytotoxic response involved in the pathogenesis of neurodegenerative diseases (Wang et al. 2020a, b).

**Fig. 7:**
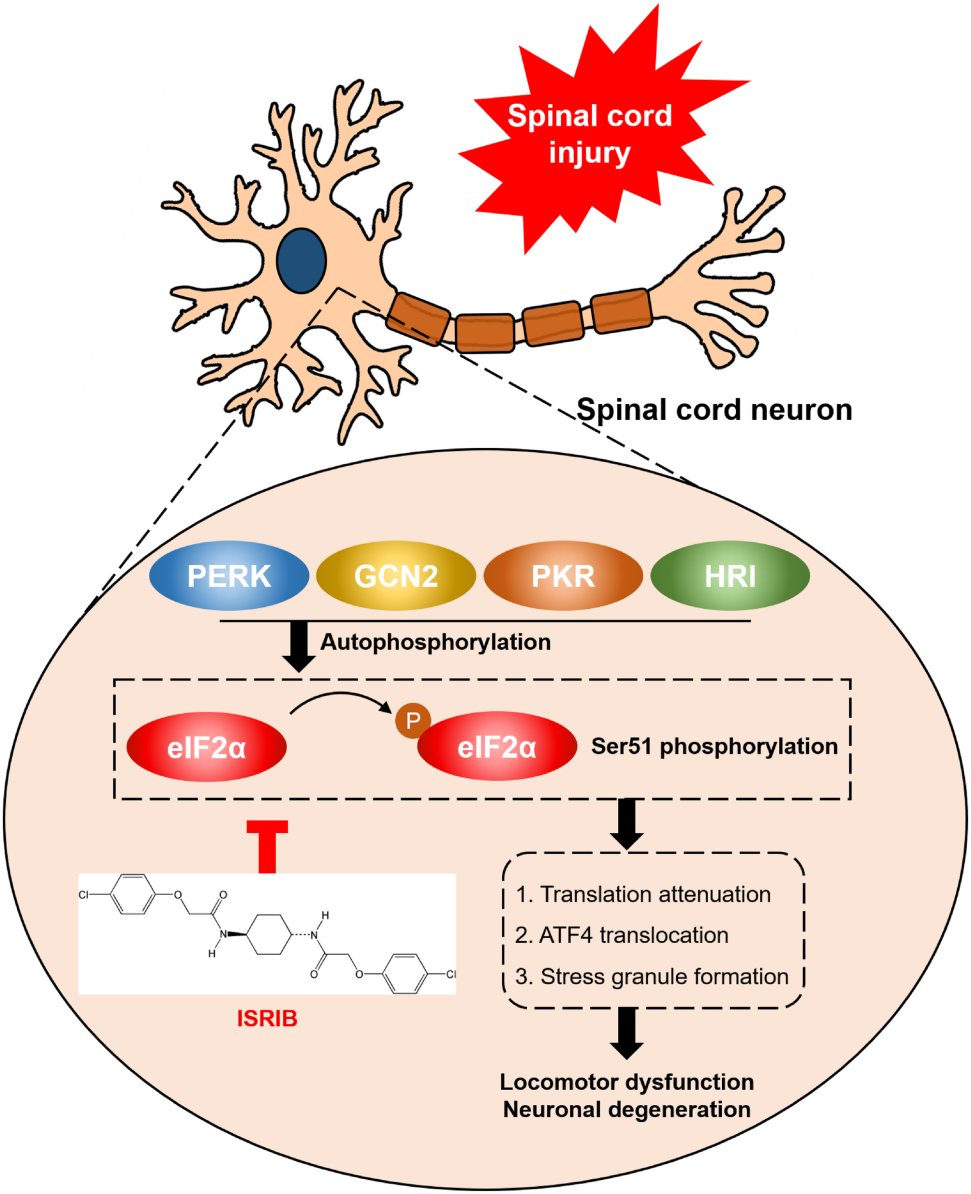
Following SCI, ISR signal is activated by autophosphorylation of PERK, GCN2, PKR, and HRI, which phosphorylates Ser51 in eIF2α to attenuate global translation and promote nuclear translocation of ATF4 and stress granule formation. Nevertheless, treatment of ISRIB after SCI alleviates ISR-induced effects to delay neuronal degeneration and improve locomotor function

Finally, we observed pathologic and functional improvement in ISRIB-treated mice subjected to SCI through H&E staining, BMS scoring, and footprint analysis. However, our research has the following limitations: (1) We solely assessed variations in neuronal fate after SCI. There are other neural cells in the CNS, and whether ISRIB directly targets neurons to exert a therapeutic effect requires further clarification. (2) The therapeutic window of ISRIB after SCI needs to be further confirmed to achieve better therapeutic results. (3) Whether ISRIB treatment has potential therapeutic significance for types of SCI other than contusion SCI, such as spinal cord hemisection and transection, remains defined. Collectively, our results confirm that the ISR is activated after SCI and prove that the cytosolic localization of RNA-binding proteins in the spinal cord, especially in neurons, is increased after SCI. Our work highlights the role of the ISR in the subacute phase after SCI and supports the use of ISRIB as a promising therapeutic agent after SCI (Fig. 7).

## Data Availability

The data that support the findings of this study are available from the corresponding author upon reasonable request.

## Abbreviations

SCI: Spinal cord injury
CNS: Central nervous system
ISR: Integrated stress response
TC: Ternary complex
UPR: Unfolded protein reaction
ERSR: Endoplasmic reticulum stress response
SGs: Stress granules
H&E: Hematoxylin & eosin
dpi: Days post injury
C-Cas3: Cleaved-caspase 3
C-Cas12: Cleaved-caspase 12
Cas12: Caspase 12
